# Asymmetric hybrid plasmonic waveguides with centimeter-scale propagation length under subwavelength confinement for photonic components

**DOI:** 10.1186/1556-276X-9-599

**Published:** 2014-11-04

**Authors:** Wei Wei, Xia Zhang, Xiaomin Ren

**Affiliations:** 1 State Key Laboratory of Information Photonics and Optical Communications, Beijing University of Posts and Telecommunications, P.O. Box 66, Beijing 100876, China

**Keywords:** Surface plasmons, Plasmonic waveguides, Optical interconnections, Integrated photonics

## Abstract

An asymmetric hybrid plasmonic metal-wire waveguide is proposed by combining the advantages of symmetric and hybrid plasmonic modes. The idea of asymmetric structure eliminates the adverse effect of a substrate and enhances the optical performance of the waveguide. The guiding properties of the proposed waveguide are intensively investigated using the finite elements method. The results exhibit a quite long propagation length of 2.69 cm with subwavelength confinement. More importantly, an extremely large figure of merit of 139037 is achieved. Furthermore, the proposed waveguides can be used as directional couplers. They can achieve a coupling length of only 1.01 μm at *S* = 0.1 μm with negligible loss. A strong dependence of coupling length on the operating wavelength makes the proposed waveguide promising for realizing wavelength-selective components at telecommunication wavelengths.

## Background

Low-level integration of integrated photonic circuits resulting from the diffraction limit of light hinders the usage of electromagnetic waves as information carriers in optical signal-processing devices and integrated photonic circuits [1,2]. Surface plasmons (SPs), which are optically induced oscillations of the free electrons at the surface of metal with negative dielectric permittivity and strongly localized at nanoscale near metal-dielectric interfaces, provide a promising solution to guiding light beyond the diffraction limit of light [3,4]. The capability of guiding light with deep subwavelength confinement is of great interest for practical applications in photonics leading to ultra-dense integration of photonic circuits [5]. However, the propagation lengths of the tightly confined modes in plasmonic waveguides are not large enough due to the presence of ohmic losses in the dissipative metal regions. The new approach to circumvent this challenge is the symmetric hybrid plasmonic (SHP) waveguide, which integrates hybrid plasmonic modes [6] and symmetric plasmonic modes (sometimes referred to as long-range plasmonic mode) [7,8] to provide a good compromise between propagation length and confinement [9-16]. For further practical implementations, an asymmetric hybrid plasmonic waveguide is proposed to overcome the influence of the substrate on the guiding properties of a symmetric hybrid plasmonic waveguide and symmetrize the broken symmetric plasmonic mode by introducing an asymmetry into the SHP waveguide [17].

In this paper, we propose an asymmetric hybrid plasmonic waveguide embedded with a metal nanowire, which is called the asymmetric hybrid plasmonic metal-wire waveguide (AHPMW) to further extend the propagation length with subwavelength confinement. Using the finite elements method (FEM), the guiding properties including mode effective index, propagation length, normalized modal area, and figure of merit (FoM) are intensively investigated at a wavelength of 1,550 nm to target potential applications in telecommunications. With optimized parameters of the AHPMW waveguide, the maximum propagation length can be 2.69 cm with subwavelength confinement. A corresponding FoM of 139037 is obtained, which is significantly larger than most previously reported ones. The proposed AHPMW waveguide also exhibits good tolerance to slight slopes in sidewalls. Moreover, the coupling properties of the AHPMW waveguides have been investigated using a full three-dimensional FEM (3D-FEM) to explore applications in directional couplers and wavelength-selective components. By tuning the separation between the AHPMW waveguides, the directional couplers can be used as wavelength-selective components operating at the telecommunication wavelength. Through optimizing the parameters of the directional couplers, the performance could be further improved to design compact photonic components with strong coupling required, such as switches, multiplexers, and wavelength-selective components.

## Methods

**Figure 1:**
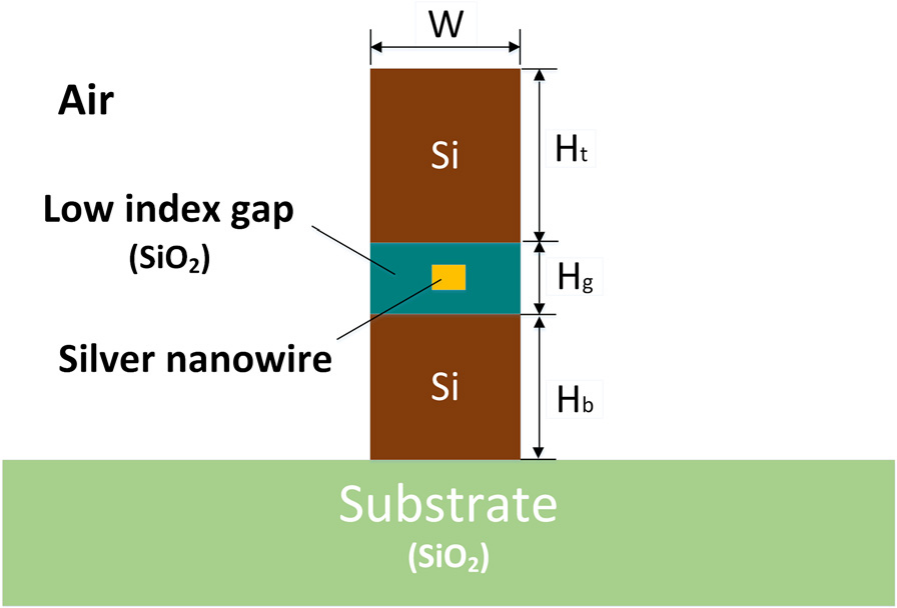
Schematic diagram of the AHPMW waveguide.

The schematic diagram of AHPMW waveguide is shown in Figure 1. It has a width of *W* = 150 nm. A silver nanowire with a side length of 5 nm is embedded in the center of the low-index dielectric gap. The height of the low-index gap is denoted by *H*_g_. The top and bottom of the waveguide are dielectric layers with high refractive indices (silicon). Silica is chosen to form the low-index gap with respect to silicon. In a SHP waveguide, *H*_t_ is equal to *H*_b_ to keep the symmetries of the mode and structure. The asymmetry of the AHPMW waveguide is generated by decreasing *H*_b_. The values of *H*_t_ and *H*_b_ are 300 and 275 nm, respectively. The mismatch between *H*_t_ and *H*_b_ breaks the symmetry of the waveguide, but it symmetrizes the mode profile along with extending the propagation length. The guiding properties of the AHPMW waveguide are investigated using FEM at 1,550 nm. The refractive index of silver is taken from [18]. Here, it is worth mentioning that the heights and widths of metal nanowires in the AHPMW waveguides considered in the study are reaching the limit where the local solutions of the macroscopic Maxwell's equations may not be accurate enough for the descriptions of the electromagnetic properties. For more rigorous investigations, one needs to take non-local effects into account [6,19,20].

**Figure 2:**
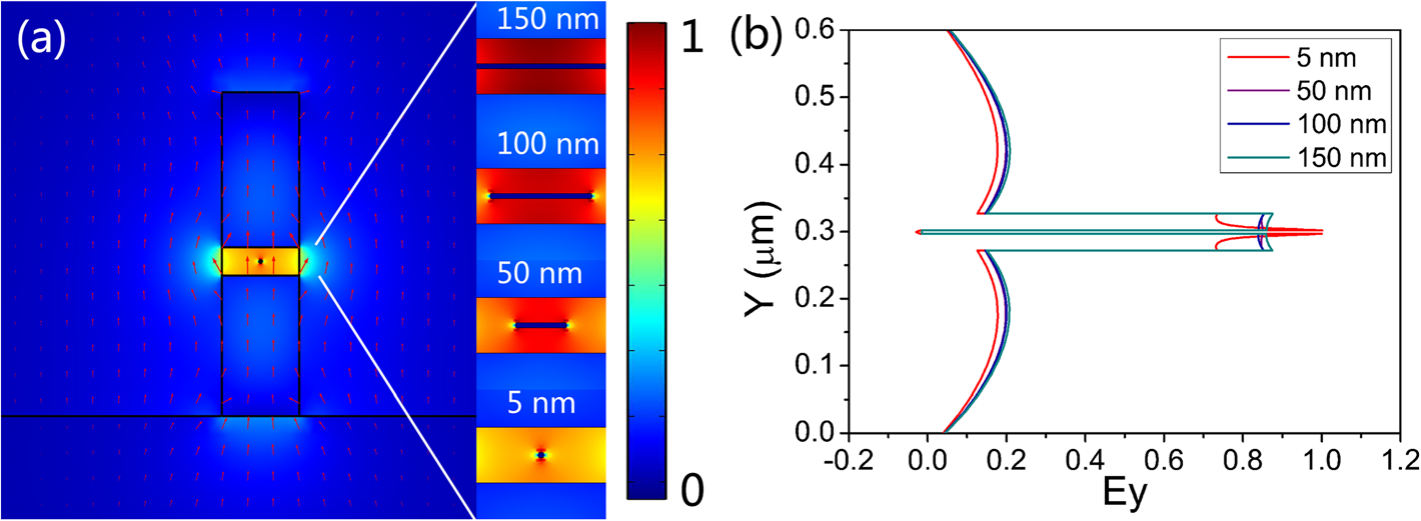
**Profiles (a) and normalized magnitude along *Y*-axis (b) of *E***_***y ***_**in the AHPMW waveguide.**

Since the symmetric hybrid plasmonic mode is quasi-TM in nature, both the transverse and vertical components of electric fields exist, but the transverse field component *E*_*y*_ dominates. So, here, we demonstrate the *E*_*y*_ profiles of the symmetric hybrid plasmonic modes in the AHPMW waveguide for the widths of the silver nanowire varying from 5 to 150 nm in Figure 2a. The majority of *E*_*y*_ is confined tightly in the low-index gap around the silver nanowire. The corresponding magnitude of normalized *E*_*y*_ along the *Y*-axis for different widths is shown in Figure 2b. The electric field component *E*_*y*_ has a symmetric distribution along the two vertical sides of the sliver nanowire. As the width of the silver nanowire decreases, more *E*_*y*_ distributes around the silver nanowire. We observe that decreasing the width of the metal significantly enhances the electric fields along with smaller modal dimensions.

## Results and discussion

### Guiding properties of the AHPMW waveguide

In this section, the guiding properties of the AHPMW are investigated in detail including the mode effective index, propagation length, normalized modal area, and FoM. To calculate the normalized modal area and propagation length of the AHPMW waveguide, we introduce Equations 1 to 3 [6]:

(1) $$A_{m}=\frac{W_{m}}{\max\left\{W\left(r\right)\right\}}=\frac{1}{\max\left\{W\left(r\right)\right\}}\iint_{\infty }W\left(r\right)d^{2}r\text{,}$$

where *W*_m_ is the total mode energy and *W*(*r*) is the energy density (per unit length flowed along the direction of propagation). For dispersive and lossy materials, the *W*(*r*) inside can be calculated using Equation 2:

(2) $$W\left(r\right)=\frac{1}{2}\left(\frac{d\left(\epsilon \left(r\right)\omega \right)}{d\omega }{\left|E\left(r\right)\right|}^{2}+\mu_{0}{\left|H\left(r\right)\right|}^{2}\right)\text{.}$$

The normalized modal area is defined as *A*_m_ / *A*_0_ to quantitatively evaluate the mode confinement, where *A*_0_ denotes the diffraction-limited mode area in free space, *A*_0_ = *λ*^2^ / 4. The propagation length is defined as Equation 3:

(3) $$L=\frac{1}{2Im\left(\beta \right)}\text{.}$$

Different plasmonic waveguides have different propagation lengths and mode confinements as well, and it is not intuitive to compare them directly due to the general rule between the two quantities in plasmonic waveguides. So, it is more convenient to propose FoM which takes into account both quantities and then one can directly use the FoM to assess a plasmonic waveguide. The FoM is defined as the ratio of the propagation length to the effective mode size, where the effective mode size is the diameter of the effective modal area [21].

(4) $$FoM=\frac{L}{2\sqrt{A_{m}/\pi }}\text{.}$$

**Figure 3:**
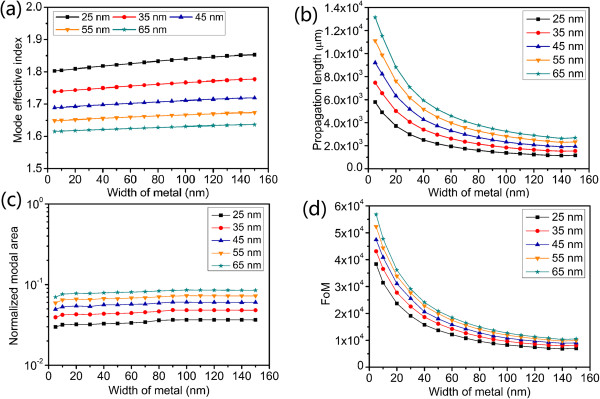
**Dependence of symmetric hybrid plasmonic mode's properties on the width of metal for different *H***_**g**_**. (a)** Mode effective index, **(b)** propagation length, **(c)** normalized modal area, and **(d)** FoM.

Dependences of the symmetric hybrid plasmonic mode's properties on the width of the metal nanowire for different gap heights are shown in Figure 3. The low-index gap symmetrically surrounds the metal nanowire. Its height varies from 25 to 65 nm including the weight of metal nanowire. In Figure 3a, the mode effective indices of the AHPMW waveguide for different gap heights increase as the width of the metal becomes wider. For a fixed width, the mode effective indices of the AHPMW waveguide have negative correlations with the heights of the low-index gap. When the height of the low-index gap decreases, the metal nanowire gets closer to the high index regions resulting in the increase of the mode effective index because of the sensitivity of SPs to the surrounding dielectrics. The two main factors of the propagation length (loss) and the normalized modal area (confinement) describing the guiding properties are presented in Figure 3b,c. With the increasing width of metal nanowire, the propagation lengths and the normalized modal areas have inverse trends. Associating with Figure 2, we find that the electric field component *E*_*y*_ is confined more tightly around the metal nanowire as the width of the metal becomes narrower. As a consequence, the normalized modal area decreases and the metal nanowire dissipates less energy, leading to a longer propagation length. For different heights of the low-index gap, both the propagation length and normalized modal area increase with the increasing height of the low-index gap. As most energy of the symmetric hybrid plasmonic mode is confined tightly within the low-index gap, when the low-index gap becomes narrower, the energy is distributed in a smaller region and more energy is confined around the metal nanowire. So, both the propagation length and the normalized modal area exhibit positive correlations with the height of the low-index gap. In Figure 3b, when the width of the metal and the height of the low-index gap are 5 and 65 nm, respectively, the propagation length presents a maximum of 1.31 × 10^4^ μm. The corresponding normalized modal area is 6.99 × 10^-2^. So, the proposed AHPMW waveguide demonstrates a centimeter-scale propagation length with subwavelength confinement. It can be explained by that when the metal film is shortened to a metal nanowire, the size of the metal decreases and the symmetric hybrid plasmonic mode is tightly confined in the low-index gap resulting in the low energy dissipation by the metal and small normalized modal area. Figure 3d presents the variations of the FoMs as a function of the width of the metal. With the increasing width of the metal nanowire and height of the low-index gap, the FoMs have opposite variation trends. The maximum FoM of 56816 is obtained when the width of the metal nanowire and the height of the low-index gap are 5 and 65 nm, respectively. A larger FoM means better performance of the AHPMW waveguide. Hence, in the following investigations, the width of the metal nanowire and the height of the low-index gap are fixed at 5 and 65 nm, respectively, as a reasonable compromise between propagation length and confinement.

### Tolerance to the width of AHPMW waveguide and the position of metal nanowire

**Figure 4:**
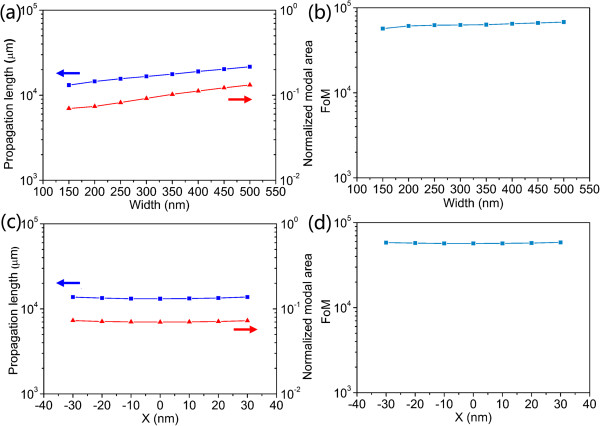
**Plasmonic properties as functions of *W *and the position of the metal nanowire.** Propagation length, normalized modal area **(a)**, and FoM **(b)** as functions of the width of the AHPMW waveguide and propagation length, normalized modal area **(c)**, and FoM **(d)** as functions of the position of the metal nanowire.

The fabrication of the structure described above includes the following steps. First, a silica layer of 2-μm thickness is coated on a silicon substrate. Then, a layer of silicon is deposited to form a silicon ridge using chemical vapor deposition (CVD) followed by a deposition of a thin film of silica on top of the silicon substrate which is precisely controlled by molecular beam epitaxy (MBE). Considering realistic large-scale manufacture, MBE can be replaced by plasma-enhanced chemical vapor deposition (PECVD) but the control of silica deposition will suffer, because deviations in the thickness of silica layer cause not much effect on the guiding properties as shown in Figure 3. Second, a silver nanowire is placed on the interior silica film. Lastly, a layer of silica is deposited on the silver nanowire followed by deposition of a silicon ridge on the silica film. Important concerns would be that a slight slope in the sidewalls and the deflected position of the metal nanowire might be harmful factors to the guiding properties of the AHPMW waveguide. The guiding properties of the AHPMW waveguide as functions of its width and the position of the metal nanowire are presented in Figure 4 to get insight into the influence of fabrication deviations on its guiding properties. Despite the asymmetric field profile along the horizontal direction (the field profile along the vertical direction is approximately symmetric), the modal characteristics remain and perform excellently with subwavelength mode confinement, reasonable propagation distance, and high FoM within a relatively wide range of geometric parameters because the metal nanowire is surrounded by the low-index gap and the modal profile is vertically symmetric, which result in tight mode confinement and natural tolerance to horizontal deviations of the AHPMW waveguide. Although a gradually increasing mode size can be observed due to a slightly weak mode confinement, its modal loss can be mitigated at the same time, resulting in extended propagation distances. This optical behavior also leads to slightly increasing FoMs. In the following sections, the width is fixed at 150 nm considering the compactness and the single-mode transportation of the AHPMW waveguides. Moreover, if in the case that the width is larger than 150 nm which results from the fabrication deviations, the performance will get better rather than worse.

### Comparison of guiding properties of AHPMW waveguides with metal nanowires featuring different cross-sectional shapes

The guiding properties of the AHPMW waveguide for four different cross-sectional shapes of the metal nanowire are summarized in Table 1. All the metal nanowires have the same height of 5 nm. The mode effective index (*n*_eff_) and normalized modal area (*A*_m_ / *A*_o_) both decrease with the increasing number of sides for different shapes (circle can be considered as a shape with infinite number of sides) except for that of triangle. This can be interpreted by that the smallest area of the triangle among the four shapes with the same height leads to the smaller normalized modal area and lower mode effective index. While for the propagation length (*L*) and FoM, they have positive correlations with the increasing number of sides for different shapes. The guiding properties of the AHPMW waveguide with circular metal nanowire perform best among the waveguides with different metal nanowires. It has a maximum propagation length of 2.69 × 10^4^ μm (2.69 cm) with strong subwavelength confinement (*A*_m_ / *A*_o_ = 4.89 × 10^-2^). More importantly, the maximum corresponding FoM of 139037 is achieved, which is significantly larger than most previously reported ones [11,14,22].

**Table 1:** The guiding properties for the four types of metal nanowires with different cross-sectional shapes

**Shape**	***n***_**eff**_	***A***_**m **_**/ *A***_**o**_	***L *** **(μm)**	**FoM**
Triangle	1.614819	6.72 × 10^-2^	1.20 × 10^4^	52825
Square	1.615327	6.99 × 10^-2^	1.31 × 10^4^	56816
Hexagon	1.614799	6.10 × 10^-2^	2.17 × 10^4^	100395
Circle	1.614470	4.89 × 10^-2^	2.69 × 10^4^	139037

**Figure 5:**
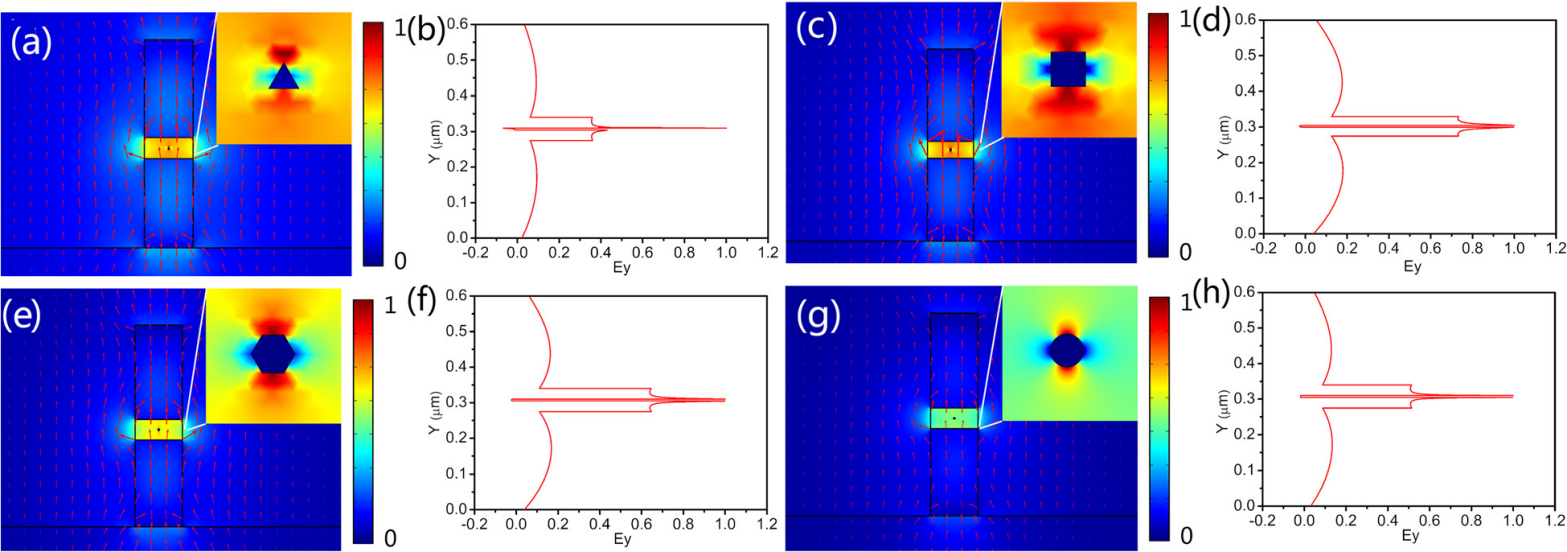
**The profiles (a)-(d) and the corresponding distributions along the *Y*-axis (e)-(h) of *E***_***y***_**.** The profiles and the corresponding distributions along the *Y*-axis of *E*_*y*_ in the AHPMW waveguide for the different transverse profiles of the metal nanowire. The red arrows indicate the orientations of *E*_*y*_.

The profiles and the corresponding magnitudes along the *Y*-axis of the electric field component *E*_*y*_ in the AHPMW waveguide for different transverse metal nanowires are shown in Figure 5. For the AHPMW waveguide with triangle metal nanowire, the electric field component *E*_*y*_ distributes asymmetrically along the *Y*-axis of the waveguide, and the major *E*_*y*_ is confined at the upper section of the triangle metal, which results in the decline of the propagation length. Whereas for the AHPMW waveguides with other transverse nanowires of metal nanowires, *E*_*y*_ distributes symmetrically along the metal nanowire, and the major *E*_*y*_ is confined in the low-index gap in contrast with the high-index regions. Thus, the AHPMW waveguide can realize quite long propagation length to a centimeter level. Among the AHPMW waveguides with four metal nanowires, the one with a circular metal nanowire exhibits the strongest confinement of *E*_*y*_ along with the longest propagation length. So, in the following investigations, a circular metal nanowire is embedded in the center of the low-index gap of the AHPMW waveguide to achieve the best performance.

### Bend radius of the AHPMW waveguide

**Figure 6:**
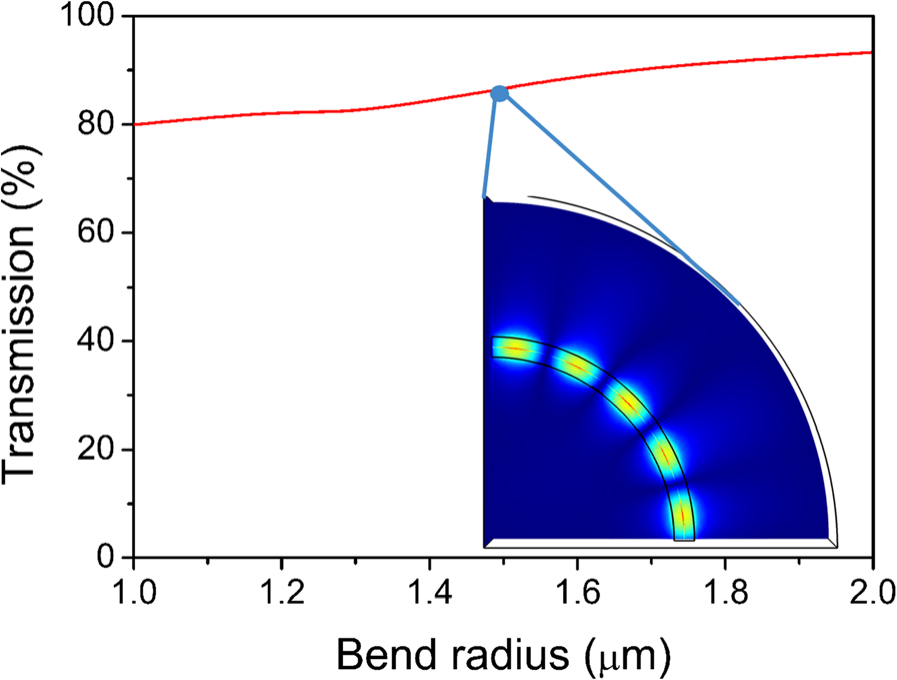
**Transmission versus bend radius.** The inset is the evolution of |E|.

In relation to high integration of photonic circuits, maintaining low radiation loss while transmitting light signals in a sharp bend is an important requirement. Power transmissions versus bend radius, which is shown in Figure 6, is an important parameter to evaluate the bending performance of the proposed AHPMW waveguide. The power transmission is defined as the ratio of the Poynting vector integral in the input plane to that in the output plane. Theoretically, the total loss includes bend loss and propagation loss due to ohmic absorption. Nevertheless, the proposed AHPMW waveguide has a quite long propagation length, namely quite low propagation loss. Thus, the calculated transmission is dominated mainly by bend loss. As the transmission shown in Figure 6, it maintains above 80% and behaves an increasing trend with the increase of the bend radius. The AHPMW waveguide demonstrates the greatest transmission of 93.3% when the bend radius is 2 μm. The inset in Figure 6 shows the evolution of |E| corresponding to the bend radius of 1.5 μm. Most |E| is tightly confined around the silver nanowire in the low-index gap of the bending AHPMW waveguide leading to the low radiation loss along with the high transmission, which is beneficial to design waveguides and devices in highly integrated photonic circuits.

### Coupling properties of the AHPMW waveguides

For applications in highly integrated photonic circuits as interconnects, examining the allowable separation of the two adjacent AHPMW waveguides with negligible crosstalk is necessary. To get further insight into the operations of coupling AHPMW waveguides, we conducted full 3D-FEM simulations, which allow us to retrieve the electric field transfer process and the coupling length dispersion.

### Directional couplers based on AHPMW waveguides

**Figure 7:**
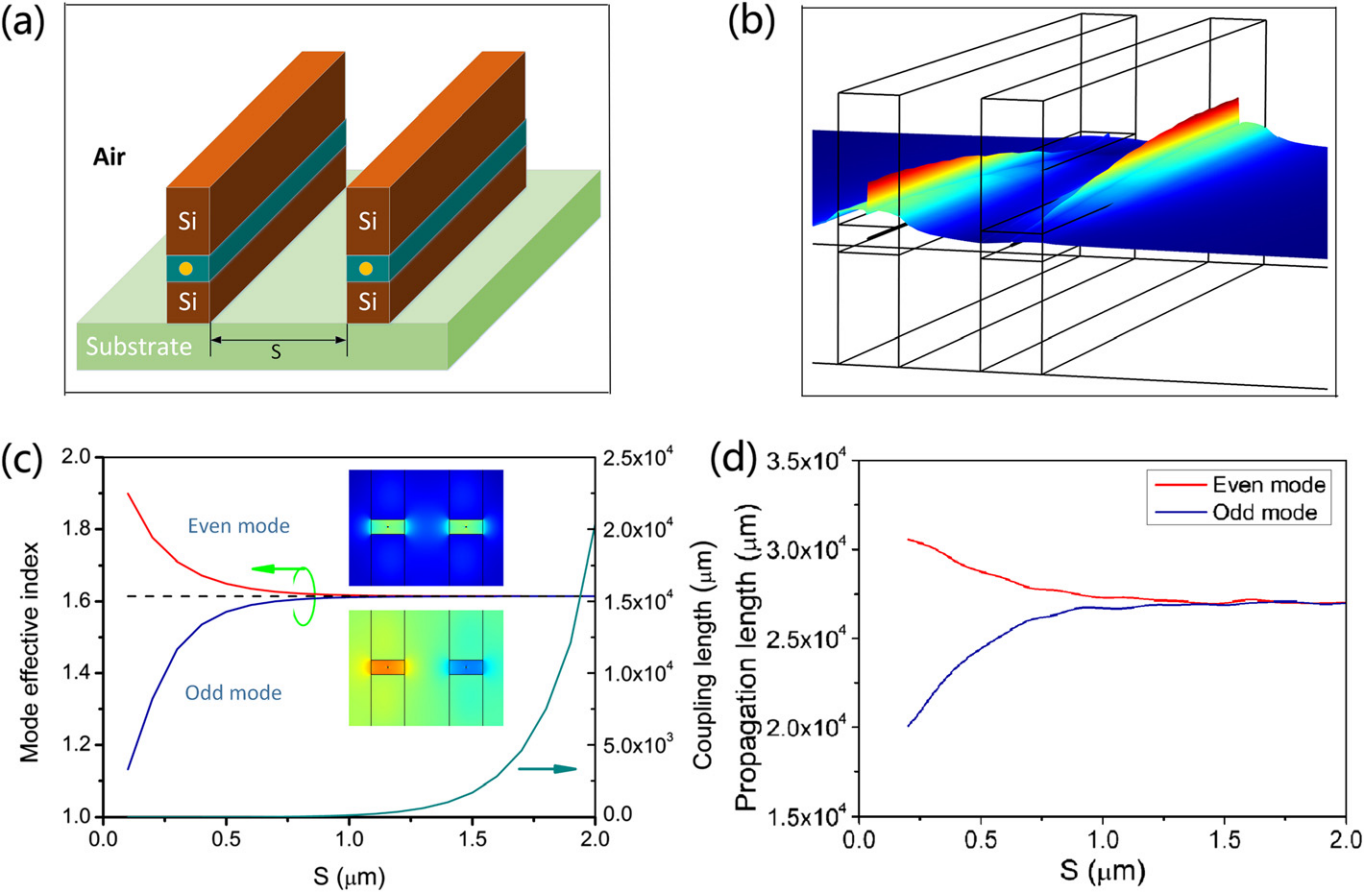
**Coupling properties of a directional coupler based on two adjacent AHPMW waveguides. (a)** Schematic of the wavelength-selective component, **(b)** the corresponding 3D |E| evolution, **(c)** dependence of the mode effective index and the coupling length on the separation, and **(d)** propagation lengths of the even and odd modes as functions of *S*.

To visualize the electric field transfer process, 3D |E| evolution of the AHPMW waveguides with *S* = 0.2 μm is demonstrated in Figure 7b. Theoretically, the directional coupler consisting of two single-mode waveguides supports an even (symmetric) mode and an odd (asymmetric) mode relative to the *E*_*y*_ field profiles. For the observed patterns, the insets in Figure 7c show the *E*_*y*_ field profiles of the even and odd modes. Based on the coupled-mode theory [23], the coupling strength is calculated by the coupling length *L*_c_ = *λ* / [2 × Re(*n*_even_ - *n*_odd_)], where *n*_even_ and *n*_odd_ denote the real parts of the mode effective indices of even and odd modes, respectively. As the difference between the imaginary parts of the mode effective index is much smaller than that between the real parts of the mode effective index for even and odd modes, the derived coupled-mode theory can still be used in lossy waveguides [24]. The dependence of the mode effective index and coupling length on the separation from 0.1 to 2 μm is presented in Figure 7c. It is observed that the coupling length varies noticeably with the separation. As the separation distance increases, the mode effective indices of the even and odd modes approach that of a single waveguide, leading to the smaller difference of mode effective indices for the even and odd modes. So, according to the equation of the coupling length, the coupling length becomes larger with the increasing separation. The phenomenon reveals that the decoupling (no crosstalk) appears when the two waveguides are sufficiently far apart. When the separation distance is smaller than 100 nm, the AHPMW waveguide cannot support the odd mode. The propagation lengths of the even and odd modes are quite long (centimeter level), as shown in Figure 7d. To design compact photonic components with strong coupling required, the directional coupler based on the AHPMW waveguides can achieve a coupling length of only 1.01 μm at *S* = 0.1 μm with negligible loss, which is approximately 26,000 times smaller than the propagation length of the AHPMW waveguide, while, the directional coupler requires the separation of 2.5 μm to achieve no coupling. The results indicate potential applications of the AHPMW waveguides in highly integrated photonic circuits as directional couplers.

### Wavelength-selective components based on the AHPMW waveguides

**Figure 8:**
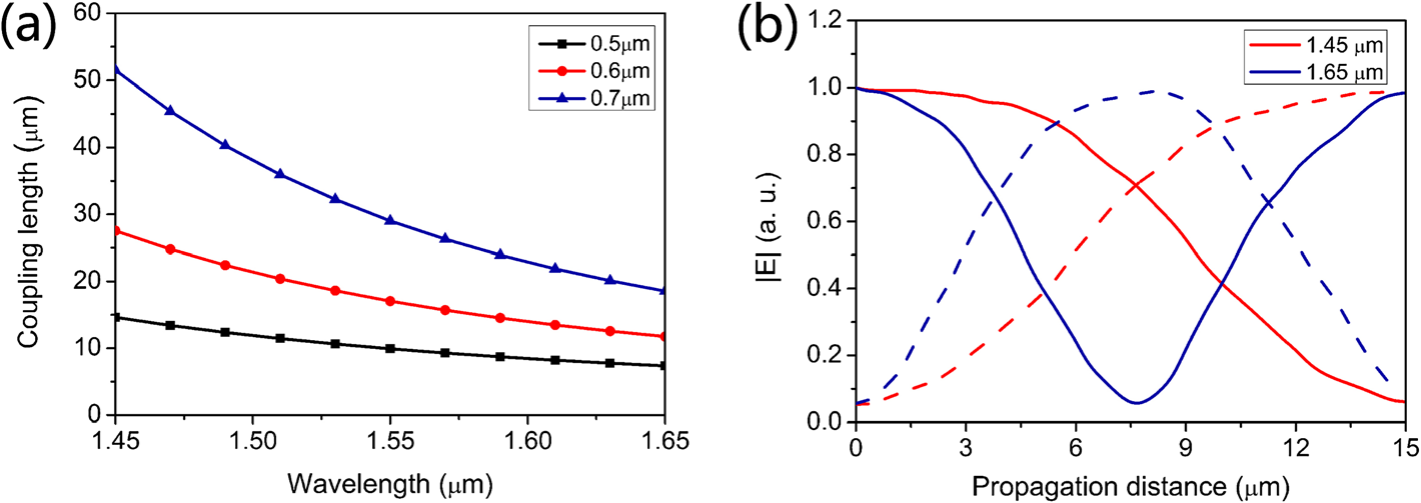
**Coupling properties of wavelength-selective component based on two adjacent AHPMW waveguides. (a)** Dependence of the coupling lengths on wavelength for different separations and **(b)** electric field |E| transfer process in two AHPMW waveguides for wavelengths of 1.45 and 1.65 μm.

To further examine the feasibility of the proposed AHPMW waveguide in designing the wavelength-selective component based on directional coupler, the dependence of the coupling length on wavelength across the range of 1.45 to 1.65 μm for three separations of *S* = 0.5, 0.6, and 0.7 μm is investigated taking into account material dispersion. As shown in Figure 8a, it is observed that the coupling length depends strongly on wavelength and there is a negative correlation between the coupling lengths and wavelength. Among the three curves, one for S = 0.7 μm possesses the fastest decrease with the increasing wavelength.To separate one wavelength from the other, the coupling length of the AHPMW waveguides for the wavelength needs to be two times than that for the other wavelength, as shown in Figure 8b. Two signals are injected into the directional coupler from the left waveguide. Then, after a propagation length of 15 μm, the signal of 1.45 μm couples to the right waveguide, while the signal of 1.65 μm couples to the right waveguide and then back to the left waveguide. Finally, signals of 1.45 and 1.65 μm output from the right waveguide and the left waveguide, respectively. Therefore, the directional couplers based on the AHPMW waveguides are adaptive to be used as the wavelength-selective components.

## Conclusions

The AHPMW waveguide embedded with a metal nanowire in the low-index gap has been proposed, which combines the symmetric and hybrid plasmonic modes to achieve quite long propagation length with subwavelength confinement. The introduced asymmetry symmetrized the asymmetric plasmonic modes along with the extension of the propagation length. Using FEM, the guiding properties of the AHPMW waveguide was theoretically analyzed at the telecommunication wavelength. The results revealed that with optimized parameters of the AHPMW waveguide, a centimeter-scale propagation length of 2.69 cm was achieved with normalized modal area of 4.89 × 10^-2^ to realize nanoscaled mode confinement. And the corresponding FoM of 139037 was achieved, which is significantly greater than most previously reported ones. In addition, the proposed AHPMW waveguide has tolerance to slight slopes in sidewalls. Furthermore, the coupling properties of the AHPMW waveguides were investigated in detail to be used as directional couplers. The directional coupler could achieve a coupling length of only 1.01 μm at *S* = 0.1 μm with negligible loss resulting in potential applications in compact photonic circuits as interconnects. Strong dependences of coupling length on both wavelength and separation make the AHPMW waveguide promising for realizing a number of plasmonic components, such as switches, multiplexers, and wavelength-selective components for the selection and spatial separation of radiation channels at different wavelengths.

## Abbreviations

AHPMW: asymmetric hybrid plasmonic metal-wire waveguide; FEM: finite elements method; FoM: figure of merit; SPs: surface plasmons.

## Competing interests

The authors declare that they have no competing interests.

## Authors’ contributions

WW proposed the waveguide structure, calculated the properties of the proposed waveguide, and wrote the manuscript. XZ and XR analyzed the data and revised the manuscript. All authors read and approved the final manuscript.

## References

[B1] Born M Wolf E Principles of Optics 1999 Cambridge: Cambridge University Press

[B2] Gramotnev DK Bozhevlnyi SI Plasmonic beyond the diffraction limit Nat Photon 2010 4 83 91 10.1038/nphoton.2009.282

[B3] Polman A Applied physics plasmonics applied Science 2008 322 868 869 10.1126/science.1163959 18988831

[B4] Belan S Vergeles S Vorobev P Adjustable subwavelength localization in a hybrid plasmonic waveguide Opt Express 2013 21 7427 7438 10.1364/OE.21.007427 23546126

[B5] William LB Alain D Thomas WE Surface plasmon subwavelength optics Nature 2003 424 824 830 10.1038/nature01937 12917696

[B6] Oulton RF Sorger VJ Genov DA Pile DFP Zhang X A hybrid plasmonic waveguide for subwavelength confinement and long-range propagation Nat Photon 2008 2 496 500 10.1038/nphoton.2008.131

[B7] Burke JJ Stegeman GI Surface-polariton-like waves guided by thin, lossy metal films Phys Rev B 1986 33 5186 5201 10.1103/PhysRevB.33.5186 9939016

[B8] Berini P Plasmon-polariton modes guided by a metal film of finite width bounded by different dielectrics Opt Express 2000 7 329 335 10.1364/OE.7.000329 19407883

[B9] Bian YS Zheng Z Zhao X Zhu JS Zhou T Symmetric hybrid surface plasmon polariton waveguides for 3D photonic integration Opt Express 2009 17 21320 21325 10.1364/OE.17.021320 19997371

[B10] Chen L Li X Wang GP Li W Chen SH Xiao L Gao DS A silicon-based 3-D hybrid long-range plasmonic waveguide for nanophotonic integration J LightW Technol 2012 30 163 168

[B11] Chen L Zhang T Li X Huang WP Novel hybrid plasmonic waveguide consisting of two identical dielectric nanowires symmetrically placed on each side of a thin metal film Opt Express 2012 20 20535 20544 10.1364/OE.20.020535 23037100

[B12] Bian YS Gong QH Low-loss light transport at the subwavelength scale in silicon nano-slot based symmetric hybrid plasmonic waveguiding schemes Opt Express 2013 21 23907 23920 10.1364/OE.21.023907 24104301

[B13] Noghani MT Samiei MHV Analysis and optimum design of hybrid plasmonic slab waveguides Plasmonics 2013 8 1155 1168 10.1007/s11468-013-9526-x

[B14] Huang CC Ultra-long-range symmetric plasmonic waveguide for high-density and compact photonic devices Opt Express 2013 21 29544 29557 10.1364/OE.21.029544 24514506

[B15] Chen L Zhang T Li X Enhanced optical forces by hybrid long-range plasmonic waveguides J LightW Technol 2013 31 3432 3438

[B16] Bian YS Gong QH Deep-subwavelength light confinement and transport in hybrid dielectric-loaded metal wedges Laser Photon Rev 2014 8 549 561 10.1002/lpor.201300207

[B17] Wei W Zhang X Huang YQ Ren XM Guiding properties of asymmetric hybrid plasmonic waveguides on dielectric substrate Nanoscale Res Lett 2014 9 13 10.1186/1556-276X-9-13 24406096 PMC3895746

[B18] Palik ED Handbook of Optical Constants of Solids 1985 New York: Academic

[B19] Garcia De Abajo FJ Nonlocal effects in the plasmons of strongly interacting nanoparticles, dimers, and waveguides J Phys Chem C 2008 112 17983 17987 10.1021/jp807345h

[B20] Marinica DC Kazansky AK Nordlander P Aizpurua J Borisov AG Quantum plasmonic: nonlinear effects in the field enhancement of a plasmonic nanoparticle dimer Nano Lett 2012 12 1333 1339 10.1021/nl300269c 22320125

[B21] Buckley R Berini P Figure of merit for 2D surface plasmon waveguides and application to metal stripes Opt Express 2007 15 12174 12182 10.1364/OE.15.012174 19547584

[B22] Jeong CY Kim M Kim S Circular hybrid plasmonic waveguide with ultra-long propagation distance Opt Express 2013 21 17404 17412 10.1364/OE.21.017404 23938588

[B23] Huang WP Coupled-mode theory for optical waveguides: an overview J Opt Soc Am A 1994 11 963 983 10.1364/JOSAA.11.000963

[B24] Veronis G Fan SH Crosstalk between three-dimensional plasmonic slot waveguides Opt Express 2008 16 2129 2140 10.1364/OE.16.002129 18542293

